# Einsatz von „continuous passive motion“ in der stationären Anschlussheilbehandlung von Schulterendoprothesen – eine retrospektive Untersuchung

**DOI:** 10.1007/s00132-026-04808-x

**Published:** 2026-03-20

**Authors:** Christoph Schulze, Jan Peters, Klara Tillack, Claudia Bünzen, Sibylle Schlüter

**Affiliations:** 1 Institut für Physikalische Medizin und Allgemeine Rehabilitation, Universitätsklinikum der Paracelsus Medizinischen Privatuniversität, Müllner-Hauptstr. 48, 5020 Salzburg, Österreich; 2 Orthopädische Klinik und Poliklinik, Universitätsmedizin Rostock, Doberaner Str. 152, 18057 Rostock, Deutschland; 3 Institut für Sportwissenschaft, Sportmedizin, Christian-Albrechts-Universität Kiel, Olshausenstraße 74, 24118 Kiel, Deutschland; 4 Dr. Ebel Fachklinik Moorbad Bad Doberan, Schwaaner Chaussee 2, 18209 Bad Doberan, Deutschland; 5 Klinik für Orthopädie und Unfallchirurgie, Plastische, Rekonstruktive und Handchirurgie, Bundeswehrkrankenhaus Westerstede, Westerstede, Deutschland; 6 Fachärztliche Untersuchungsstelle für Orthopädie und Unfallchirurgie, Facharztzentrum der Bundeswehr in Augustdorf, Augustdorf, Deutschland

**Keywords:** Gelenkersatz, Mobilisierung, Schmerzwahrnehmung, Postoperative Versorgung, Schultergelenk, Joint replacement, Mobilization, Pain perception, Postoperative care, Shoulder joint

## Abstract

**Hintergrund:**

Die Anwendung von „continuous passive motion“(CPM)-Geräten findet breite Anwendung im Bereich der Behandlung von Gelenkerkrankungen. Während der Einsatz von CPM nach Implantation von Knieendoprothesen bereits gut untersucht ist, fokussiert diese Studie die Nutzung von Bewegungsschienen nach Implantation einer Schulterendoprothese.

**Material und Methoden:**

In dieser retrospektiven Auswertung wurden 110 Patienten auf Veränderungen in der Schmerzwahrnehmung und Schmerzmitteleinnahme untersucht. Bei 45 Patientendatensätzen konnten die Entwicklung des aktiven Bewegungsausmaßes und die Vorführbarkeit von Schürzen- und Nackengriff nach Durchführung regelmäßiger CPM-Behandlung im Rahmen einer komplexen Anschlussheilbehandlung nach Implantation einer Schulterendoprothese untersucht werden. Zusätzlich wurde der mögliche Einfluss des Startzeitpunktes der CPM-Behandlung statistisch mittels Wilcoxon-Test für verbundene Stichproben und Korrelationsanalyse nach Pearson untersucht.

**Ergebnisse:**

Bei der Anwendung von CPM als Komponente im rehabilitativen Behandlungskonzept konnte eine Reduktion der Schmerzwahrnehmung und des Schmerzmittelbedarfs, sowie eine Verbesserung des Bewegungsausmaßes festgestellt werden. Ein signifikanter Zusammenhang mit dem zeitlichen Beginn der Therapie und dem Ergebnis am Ende der Rehabilitation konnte nicht gezeigt werden, aber die Anwendungshäufigkeit korrelierte positiv mit der Veränderung des Bewegungsausmaßes.

**Schlussfolgerung:**

Die Ergebnisse dieser Studie weisen auf einen positiven Effekt der CPM-Schienentherapie nach Implantation einer Schulterendoprothese im Rahmen der Rehabilitation hin. Aufgrund des Studiendesigns ohne Kontrollgruppe ist eine klare Trennung zwischen einem Rehabilitationsverlauf ohne Intervention, dem Effekt einer multimodalen Therapie und dem spezifischen Effekt der CPM-Therapie nicht möglich. Welchen Anteil am beobachteten Effekt auf die CPM-Schienentherapie zurückzuführen ist und welche eventuellen Vorteile gegenüber einer manuellen Mobilisierung oder einer rein funktionalen Beübung bestehen, kann nicht beantwortet werden. Dieses und auch Aspekte der Wirtschaftlichkeit sollten in weiteren Studien überprüft werden.

**Graphic abstract:**

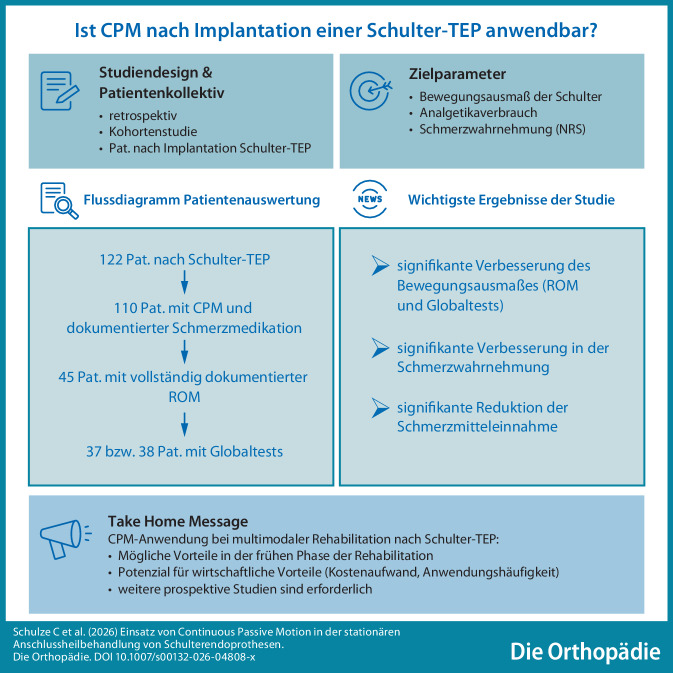

## Abstract (en)

**Background:**

Continuous passive motion (CPM) devices are widely used in the therapy of joint diseases. While the use of CPM after arthroplasty of the knee is already well evaluated, this study focuses on the use of CPM-devices after shoulder arthroplasty.

**Methods:**

In this retrospective analysis, 110 patients were examined for changes in pain perception and use of pain medication. For 45 of them, the development of the active range of motion and the ability to perform the superior and inferior Apley scratch test were investigated after regular CPM treatment as part of a multimodal rehabilitation program following shoulder arthroplasty. Additionally, the potential influence of the initiation time of CPM treatment was statistically analyzed using the Wilcoxon signed-rank test for paired samples and Pearson’s rank correlation analysis.

**Results:**

Using CPM as a component in a rehabilitation treatment concept led to a reduction in pain perception and the need for pain medication. Furthermore, an improvement in the range of motion, was observed. A significant correlation between the timing of the therapy’s initiation and the outcome at the end of rehabilitation could not be demonstrated, but the frequency of application correlated positively with changes in the range of motion.

**Conclusion:**

The results of this study imply potential for benefits from the use of CPM devices after shoulder replacement surgery as part of rehabilitation. Due to the study design without any control group, a clear distinction between the rehabilitation without intervention, the effect of a multimodal therapy, and the specific effect of CPM therapy is not possible. Furthermore, it is not possible to determine what effect is attributable to CPM therapy and what potential advantages may exist, compared to manual mobilization or purely functional exercises. These questions, as well as aspects of cost-effectiveness, should be investigated in further studies.

## Einleitung

Die Arthrose des Glenohumeralgelenks ist mit einer Inzidenz von 33 % bei über 60-Jährigen in der Bevölkerung aktuell noch seltener vertreten als die Arthrose im Knie- oder Hüftgelenk [5, 10]. Dabei stieg die Anzahl der im Prothesenregister der Deutschen Vereinigung für Schulter und Ellenbogenchirurgie erfassten Prothesen bis 2019 stetig an [4]. 2021 wurden in Deutschland geschätzt etwa 25.000 Schulterendoprothesen implantiert [17]. Damit konnte in diesem Bereich der Endoprothetik der vergleichsweise stärkste Zuwachs bezüglich der Zahl der Implantationen verzeichnet werden. Der Stand der heutigen Prothesentechnik ist jedoch einem stetigen Wandel und einer langen Geschichte der Entwicklung verschiedener Generationen von Prothesentypen und Operationstechniken zuzuschreiben. So wurde die schon 1893 entwickelte erste gekoppelte Prothese von Charles Neer zur heute immer noch verwendeten anatomischen Totalendoprothese Neer‑2 weiterentwickelt und 1985 auch durch die inverse Schulterendoprothese von Grammont ergänzt [17]. Aktuell verwendete Modelle unterscheiden sich zusätzlich neben der Unterteilung in Hemi- und Totalendoprothesen bezüglich des Prothesendesigns und der Verankerung. So finden sich zum Beispiel knochensparende schaftfreie Varianten und zementierte sowie zementfreie Verankerungstypen [17].

Ebenso entscheidend für den Erfolg der Operation ist jedoch eine gut strukturierte und individuell angepasste Rehabilitation im Anschluss an die operative Versorgung [2]. Die Rehabilitationsrichtlinien diesbezüglich werden noch kritisch hinterfragt und unterliegen ständiger Aktualisierung [8, 11]. Demnach besteht eine enge Verbindung zwischen Gewinn beziehungsweise Erhalt des Bewegungsumfanges und der Weichgewebsheilung. Hierbei kann eine zu vorsichtige Behandlung eine Gelenksteife begünstigen, während eine zu aggressive Nachbehandlung insbesondere das Risiko einer beeinträchtigten Subskapularis-Sehnen-Heilung und Herabsetzung der Stabilität und Funktion der Schulter birgt [8].

Eine Möglichkeit zur exakten Einhaltung eines vorgegebenen Bewegungsumfanges und bereits fester Bestandteil von Rehabilitationsprogrammen in der Nachbehandlung verschiedener Gelenkpathologien auch an der Schulter bietet dabei die Anwendung der „Continuous passive motion“(CPM)-Technik [1]. Diese wird nach arthroskopischen Schulteroperationen häufig erfolgreich eingesetzt und der Nachweis einer Effektivität konnte bei verschiedenen Indikationen geführt werden [14]. Der Einfluss einer CPM-Schiene auf den Rehabilitationsverlauf und -erfolg nach Schulterendoprothesenversorgung wurde bislang noch nicht dezidiert untersucht, obwohl die Anwendung in publizierten Nachbehandlungsempfehlungen Erwähnung findet [13]. Gut untersucht ist der Einfluss der CPM-Schiene auf das Outcome nach Versorgung mit Knietotalendoprothesen [15]. Hier ergab sich eine Relevanz insbesondere in der frühen postoperativen Phase [18]. Neben kurzfristigen Effekten auf die Schmerzwahrnehmung konnten auch langfristige Verbesserungen des Bewegungsumfangs und des funktionellen Ergebnisses erzielt werden [9]. Obwohl bei Knietotalendoprothesen immer noch kontrovers diskutiert, wird die CPM-Anwendung hier im klinischen Alltag mit unterschiedlichsten Anwendungsparametern bereits seit vielen Jahren eingesetzt. So werden auch aktuell Studien im Hinblick auf eine Erweiterung des Einsatzbereiches der CPM-Schiene bei bisher noch nicht untersuchten Krankheitsbildern mit vielversprechenden Ergebnissen durchgeführt. Es wurden neue potenzielle Anwendungsindikationen im Bereich von Ellenbogenkontrakturen und Tibiakopffrakturen aufgezeigt [7, 12]. Diese weisen auf eine schnellere Erholung und größeren Bewegungsumfang der Gelenke nach einem Jahr mit deswegen höherer Chance auf Wiederherstellung der gewünschten Funktion hin [12]. Mögliche Bedeutungen, vor allem auch des sozioökonomischen Aspektes der Verkürzung der Dauer der Arbeitsunfähigkeit, aber auch einer möglichen Kostenreduktion durch Einsparung von Personal zur Mobilisierung, rechtfertigen eine weitere Untersuchung des Einsatzes von CPM für verschiedene Bereiche des Bewegungsapparates, insbesondere auch der Schulter [14]. Obwohl die Anwendung nach Schulterendoprothetik bereits breit angewendet wird, fehlen insbesondere hier bisher aussagekräftige indikationsbezogene Studien, wie sie im Bereich der Knieendoprothetik bereits vorliegen [15].

Ziel der Arbeit war es daher, zunächst retrospektiv zu prüfen, ob sich bei Einsatz von CPM im komplexen Behandlungskonzept einer stationären Anschlussheilbehandlung auch nach Implantation einer Schulterendoprothese eine positive Entwicklung des Bewegungsausmaßes und der Funktion der Schulter nachvollziehen lässt.

## Material und Methoden

### Patienten

**Abb. 1:**
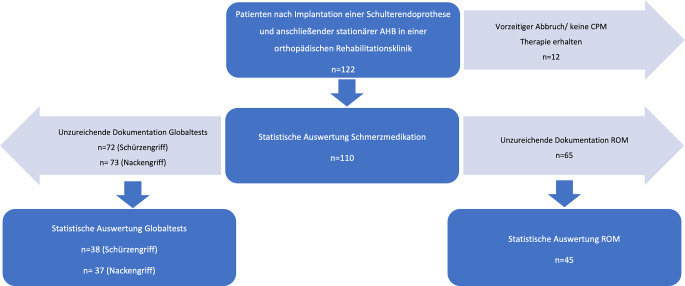
Flussdiagramm Patientenauswertung. *AHB* Anschlussheilbehandlung, *CPM* „continuous passive motion“, *ROM* „range of motion“

In diese retrospektive Kohortenstudie konnten zunächst 122 Patientendatensätze (34 männlich, 88 weiblich, Alter: 71,8 ± 10,2, BMI: 29,5 ± 5,7) einbezogen werden. Eingeschlossen wurden zunächst alle Patienten, die zwischen 2015 und 2022 nach Implantation einer Schultergelenksendoprothese eine stationäre Anschlussheilbehandlung in einer orthopädischen Rehabilitationsklinik durchführten. Als Ausschlusskriterium zählten dann die fehlende Applikation von CPM während des Rehabilitationsaufenthaltes, der vorzeitige Abbruch der Rehabilitation, sowie die fehlende Dokumentation des Bewegungsausmaßes in mehr als einer Ebene bzw. der Vorführbarkeit von Schürzen- und Nackengriff (Abb. 1). Die Patienten begannen durchschnittlich 37 Tage nach der Operation das Rehabilitationsprogramm, welches durchschnittlich 22,3 ± 4,2 Tage dauerte. Von den Patienten wurden 86 (70 %) mit einer inversen, 27 (22 %) mit einer anatomischen und 9 (7 %) mit einer Hemiendoprothese versorgt. Als Operationsursache fand sich bei 41 Patienten (33 %) ein unmittelbar vorangegangener traumatischer Auslöser, bei 65 Patienten (52 %) eine primäre Arthrose des Glenohumeralgelenks und bei 16 Patienten (13 %) eine sekundäre Arthrose. Der Studie wurde von der lokalen Ethikkommission ein zustimmendes Votum erteilt (Aktenzeichen A 2021-0119).

### Intervention

Neben der absoluten Häufigkeit und Therapiefrequenz pro Tag der CPM-Therapie wurden weitere therapeutische Maßnahmen erfasst: Einzel- und Gruppenphysiotherapie, Ergotherapie, Massage, Teilgüsse, medizinische Trainingstherapie, Reizstrom, Wassertherapie, Moorbäder, Koordinationstraining, Lymphdrainage, Fahrradtraining, Rotlicht, Hydrojet, Motoriktraining, Ultraschall, Traktionstherapie, Walking, Iontophorese, Manuelle Therapie, Moortreten und Dehnung. Des Weiteren wurden auch die ergänzenden Therapiemaßnahmen erfasst: progressive Muskelrelaxation, psychologische Einzelberatung, Schmerzbewältigung und autogenes Training.

### Datenerhebung

Die Tab. 1 zeigt die Daten, die gemäß Studienplanung erhoben werden sollten. Diese wurden aus den vorliegenden Patientenakten in Tabellen im Programm Microsoft Excel (Version 16.78, Microsoft, Redmond, WA, USA) entsprechend der Zielkriterien eingepflegt. Nach Pseudonymisierung wurden diese elektronisch gespeichert. Der aktive Bewegungsumfang am Anfang und am Ende der rehabilitativen Maßnahme wurde aus der ärztlichen, physiotherapeutischen und ergotherapeutischen Dokumentation entnommen. Dazu wurden aktive Anteversion, Retroversion, Abduktion, Adduktion, Innenrotation und Außenrotation soweit in den Unterlagen ersichtlich erhoben und jeweils die Differenz zwischen Beginn und Ende der Therapie berechnet. Zusätzlich wurde die Durchführbarkeit des Nacken- und Schürzengriffs zu Beginn und am Ende der Maßnahme erfasst. Aus den Entlassungsbriefen der Akutkliniken konnte die Verordnungshäufigkeit einer CPM-Schiene für zu Hause im Zeitraum zwischen Operation und Rehabilitation erfasst werden.

**Tab. 1:** Erhobene Daten

Patientengruppe	Patienten mit Implantation einer Schulterendoprothese und regelmäßig applizierter CPM-Therapie
Demografische Daten	Geschlecht (m/w), Alter in Jahren
Spezifische Daten	Abstand Operation – Reha-Beginn in Tagen, Aufenthaltsdauer Reha in Tagen, Therapietage effektiv, Prothesendesign (invers, anatomisch, Hemi), Häufigkeit der CPM-Anwendung (Behandlungstage, Behandlungsfrequenz am Tag), Operationsursache (Trauma, primäre oder sekundäre Arthrose)
Potenzielle Confounder	Begleiterkrankungen, wie z. B. chronische Schmerzkrankheit, Arthrosen anderer Gelenke, degenerative Wirbelsäulenveränderungen
Zielgrößen (PROM)	Veränderung der Schmerzwahrnehmung (numerische Analogskala), Veränderungen im Analgetikaverbrauch NSAR/Opiate, Veränderung im aktiven Bewegungsausmaß, Durchführbarkeit Schürzen- und Nackengriff
Bezugszeitpunkt der Erhebung	Baseline: Beginn der Rehabilitationsmaßnahme Endpunkt: Abschluss der Rehabilitationsmaßnahme

*CPM* „continuous passive motion“, *NSAR* nichtsteroidale Antirheumatika, *PROM* „Patient-reported outcome measures“

Der Opioidgebrauch zu Beginn und zum Ende der Maßnahme wurde zur besseren Vergleichbarkeit in Morphinäquivalenzdosen umgerechnet und auch für die nichtsteroidalen Antirheumatika konnten in der Wirksamkeit vergleichbare Stoffmengenäquivalenzen bilanziert werden.

### Statistische Auswertung

Die deskriptive Darstellung der Daten erfolgte bei kontinuierlichen Variablen anhand der Mittelwerte, des Medians und der Spanne. Bei kategorialen Daten wurden absolute und relative Häufigkeitsverteilungen dargestellt. Nach Prüfung auf Normalverteilung mittels Shapiro-Wilk-Test sowie grafisch mithilfe des Q‑Q Plots, wurde aufgrund der fehlenden Normalverteilung der Wilcoxon-Test für verbundene Stichproben zur Analyse einer Verbesserung in der aktiven Beweglichkeit durchgeführt. Bei der Entwicklung des Schmerzes und des Schmerzmittelkonsums wurde bei vorliegender Normalverteilung der T‑Test für verbundene Stichproben genutzt. Zur Beurteilung der Effektstärke wurde der Pearson-Korrelationskoeffizient berechnet und wie folgt interpretiert: r < 0,1 kleiner Effekt, r = 0,3 < 0,5 mittlerer Effekt und r > 0,5 starker Effekt. Die Entwicklung der Durchführbarkeit von Schürzen- und Nackengriff zu Beginn und am Ende der Reha wurde mittels McNemar-Test geprüft. Zur Überprüfung der Korrelation von metrischen Variablen wurde der Pearson-Korrelationskoeffizient herangezogen. Das Signifikanzniveau wurde auf α = 5 % (*p* ≤ 0,05) festgelegt. Alle Analysen wurden mit der Statistiksoftware IBM SPSS Statistics 23.0 (SPSS, Chicago, IL, USA) durchgeführt.

## Ergebnisse

### Schmerz

Hinsichtlich der Entwicklung des Schmerzniveaus in der Gesamtkohorte (*n* = 110) zeigten sich bei Erfassung der NRS-Skala und der dokumentierten Nutzung von nichtsteroidalen Antirheumatika signifikante Verbesserungen zwischen den Werten zu Beginn und am Ende der Reha (Tab. 2). Dabei konnte eine negative Korrelation zwischen Häufigkeit der Anwendung von CPM und Schmerzmitteleinnahme ermittelt werden (r = −0,242; *p* = 0,011). Bei Patienten, bei denen Opioide in der Schmerztherapie genutzt wurden, konnten die Äquivalenzdosen allerdings nicht signifikant reduziert werden. Bezüglich Schmerzentwicklung oder Bedarf an Schmerzmedikation konnte kein Zusammenhang mit Alter und Gewicht ermittelt werden. Auch für die Art der implantierten Endoprothese, unterteilt nach inverser, anatomischer und sonstigen Prothesenmodellen, konnte kein Einfluss auf die Schmerzentwicklung dargestellt werden.

**Tab. 2:** Outcome Schmerzmedikation zu Beginn und am Ende der Reha-Maßnahme

	Beginn (*N*)	Abschluss (*N*)	MD	*p*-Wert
Schmerz NRS-Skala ± SD	3,5 ± 2,1 (97)	2,2 ± 2,3 (25)	−1,3	0,003
Regelmäßige Schmerzmedikation in Opioidäquivalenzdosen ± SD	6,0 ± 21,0 (109)	5,1 ± 19,7 (110)	−0,9	0,564
Regelmäßige Schmerzmittel NSAR in Äquivalenzdosen ± SD	0,26 ± 0,38 (110)	0,18 ± 0,34 (110)	0,08	0,004

*N* Fallzahl, *MD* mittlere Differenz, *NRS* Numerische Rating-Skala, *NSAR* nichtsteroidale Antirheumatika, *SD* Standardabweichung

### Bewegungsausmaß

Die 45 Patienten, bei denen die Entwicklung des Bewegungsausmaßes ausreichend nachvollzogen werden konnte, waren durchschnittlich 73 Jahre alt, begannen 36,7 Tage nach der Operation das Rehabilitationsprogramm, welches im Durchschnitt 22 Tage geplant wurde und effektiv 16 Therapietage andauerte. Von diesen Patienten wurden 34 (75,6 %) mit einer inversen, 8 (17,8 %) mit einer anatomischen und 3 (6,7 %) mit einer Hemiendoprothese versorgt. Als Operationsursache fand sich bei 14 Patienten (24,4 %) ein unmittelbar vorangegangener traumatischer Auslöser, bei 26 Patienten (57,8 %) eine Arthrose des Glenohumeralgelenks und bei 5 Patienten (11,1 %) eine Arthrose auf der Grundlage von vorbelastenden Faktoren. Unter der Anwendung von CPM im Rahmen der Rehabilitationsbehandlung konnten bei allen Bewegungsrichtungen Verbesserungen in Median und Mittelwert des aktiven Bewegungsausmaßes beobachtet werden. Die Verbesserungen waren für alle Ebenen, insbesondere für die Anteversion und Abduktion signifikant (Tab. 3).

**Tab. 3:** Veränderung des aktiven Bewegungsausmaßes („range of motion“ [ROM]) in Grad

	Veränderung des aktiven ROM in Grad				
	M	MD	Spanne	*p*-Wert	Effektstärke r
Anteversion (*n* = 44)	+18,1°	+15°	−10° bis 55°	< 0,001	0,79
Retroversion (*n* = 43)	+6,9°	+5°	−40° bis 35°	< 0,001	0,56
Abduktion (*n* = 45)	+14,3°	+10°	0 bis 50°	< 0,001	0,76
Adduktion (*n* = 44)	+6,8°	+5°	−5° bis 30°	< 0,001	0,71
Außenrotation (*n* = 30)	+6,5°	+5°	−20° bis 50°	0,006	0,50
Innenrotation (*n* = 27)	+8,2°	0°	−10° bis 60°	0,008	0,51

*n* Fallzahl, *M* Mittelwert, *MD* Median

### Globaltests

Zusätzlich konnten Schürzen- und Nackengriff bei Anwendung von CPM am Ende der stationären Rehabilitation signifikant häufiger durchgeführt werden als zu Beginn (Tab. 4). Ein Einfluss des Prothesentyps auf die Entwicklung des Bewegungsausmaßes konnte in dieser Kohorte nicht festgestellt werden.

**Tab. 4:** Vorführbarkeit von Schürzen- und Nackengriff nach Schulterprothesenoperation

	Beginn Reha	Abschluss Reha	*p*-Wert
Schürzengriff (*n* = 38)	Ja = 4 (10,5 %) Nein = 34 (89,5 %)	Ja = 10 (26,3 %) Nein = 28 (73,7 %)	0,031
Nackengriff (*n* = 37)	Ja = 8 (21,6 %) Nein = 29 (78,4 %)	Ja = 16 (43,2 %) Nein = 21 (56,8 %)	0,008

### Zeitpunkt der Intervention mit CPM

Retrospektiv konnte kein signifikanter Zusammenhang zwischen dem Beginn der Intervention mit CPM im Rahmen einer Verordnung der CPM-Bewegungsschiene für zu Hause zwischen Operation und Rehabilitation auf das Ausmaß des Bewegungsumfangs zum Start oder zum Ende der Rehabilitation oder auf das Schmerzniveau bzw. den Schmerzmittelverbrauch nachgewiesen werden. Es zeigte sich aber generell ein Zusammenhang dahingehend, dass ein früherer Beginn der Reha-Maßnahme mit einem besseren Outcome hinsichtlich des Bewegungsausmaßes (z. B. Anteversion r = −0,235; *p* = 0,046) verbunden war.

## Diskussion

Die CPM wird in der Nachbehandlung von Kniegelenksendoprothesen, aber auch von operativen Versorgungen von Schulterverletzungen erfolgreich angewendet [14, 18]. Nachbehandlungsempfehlungen bei Implantation von Schulterendoprothesen sprechen sich ebenfalls für die Durchführung von CPM aus [13]. Hierfür besteht bisher noch keine Evidenz für den indikationsbezogenen Nutzen der Anwendung.

In der durchgeführten retrospektiven Kohortenstudie zeigten sich Hinweise, dass bei Anwendung von CPM im Rahmen eines multimodalen Rehabilitationsprogramms eine Verbesserung hinsichtlich Bewegungsausmaß und Schmerzempfinden erreicht werden kann. Es zeigte sich aber auch, dass insbesondere Äquivalentdosen von Morphinpräparaten nicht signifikant reduziert werden konnten, was auf mögliche Hemmnisse oder Begleiterscheinungen, wie z. B. eine Schmerzerkrankung hinweist, die unabhängig von der Nachbehandlung der endoprothetisch versorgten Schulter therapiert wurden. Postoperativ applizierte NSAR konnten hingegen reduziert werden. Die Reduktion von NSAR vor Morphinreduktion müsste gemäß WHO-Stufenmodell der Schmerzbehandlung kritisch hinterfragt werden, wenn nicht auch die Behandlung einer Schmerzerkrankung erfolgte [16]. Nebenerkrankungen, die Schmerzen verursachen (Tab. 1), müssen hier als mögliche Confounder berücksichtigt werden. Aufgrund der geringen Fallzahl, der Nutzung von Untersuchungsergebnissen verschiedener Professionen und Untersucher sowie der fehlenden Kontrollgruppe ist die Aussagekraft limitiert und ein kausaler Zusammenhang kann nicht abgeleitet werden. Gleiches limitierte die Auswertung hinsichtlich der Unterschiede im Bewegungsausmaß verschiedener Prothesenmodelle. Die positiven Effekte auf Bewegungsausmaß und Schmerz sind im Rahmen einer multimodalen Therapie mit teilweise sehr heterogenen Therapieplänen generell auch nicht ursächlich allein auf eine Maßnahme zurückzuführen. Da dies aber auf alle Einzelanwendungen im Rahmen einer Komplexbehandlung zutrifft, ist diese Limitation retrospektiv schwer zu eliminieren.

Eine breite Anwendung von CPM im Rahmen der Rehabilitation erscheint mit geringerem Personaleinsatz als für eine physiotherapeutisch begleitete Mobilisierung möglich [3]. Wirtschaftliche Vorteile wurden im Rahmen der Behandlung von Rotatorenmanschettenverletzungen bereits kontrovers diskutiert [14]. Im Rahmen dieser retrospektiven Untersuchung konnte jedoch keine Kosten-Nutzen-Abwägung erfolgen.

Die Tatsache, dass in dieser Studie Patienten von einem früheren Beginn der komplexen AHB-Maßnahme profitierten, lässt aber indirekt auch einen Schluss dahingehend zu, dass die Anwendung von CPM im Rahmen der Rehabilitationsmaßnahme frühzeitig begonnen werden kann.

Zwar konnten im Vergleich von Patienten mit und ohne vorher durch die entlassende Klinik angeordnete Heimbehandlung mittels CPM keine Unterschiede hinsichtlich des Bewegungsausmaßes und des Schmerzniveaus zu Beginn und am Ende der AHB-Maßnahme identifiziert werden, aber eine Aussage über den Erfolg einer Heimanwendung kann aus diesem Studiendesign mit dieser Fallzahl nicht unmittelbar abgeleitet werden, da insbesondere zur Qualität der Umsetzung dieser Empfehlung keine Informationen vorliegen. Andere Autoren, die Patienten mit und ohne mobilisierende Nachbehandlung beobachteten, konnten nach einem Jahr keinen Unterschied bei Patienten der unterschiedlichen Behandlungsgruppen ausmachen [6]. Demnach wäre eine wesentliche Frage, ob die maximal erreichbare Beweglichkeit mittels Anwendung einer gezielten mobilisierenden Therapie, beispielsweise mit CPM, schneller erreicht werden könnte, was eventuell Auswirkungen auf die Reduktion von Folgekosten haben könnte. Dieser Frage kann aber in dieser retrospektiven Beobachtung nicht adäquat nachgegangen werden, und die erhobenen Daten können lediglich einen Hinweis darauf geben, dass auch diese Fragestellung gezielt weiter prospektiv untersucht werden sollte, da bei Anwendung in der Nachbehandlung von Patienten mit Implantation von Endoprothesen an anderen Gelenken durchaus positive Effekte in der Frühphase berichtet wurden [18]. Hierbei sollte ebenfalls beachtet werden, dass eine Mobilisation nur so erfolgen sollte, dass das operative Ergebnis hinsichtlich der Weichteilsituation nicht gefährdet wird [8].

Zusammenfassend zeigt diese retrospektive Erhebung, dass der Einsatz der Methode CPM in der Nachbehandlung von Schulterendoprothesen im Rahmen einer Anschlussheilbehandlung an der positiven Entwicklung von Bewegungsausmaß und Schmerzniveau beteiligt sein könnte. Negative Entwicklungen konnten im Wesentlichen nicht beobachtet werden, sodass einer weiteren Untersuchung der Anwendung der Methode CPM hinsichtlich klinischer und wirtschaftlicher Vor- oder Nachteile in der postoperativen Heimbehandlung und der Anschlussheilbehandlung nichts entgegensteht.

## Schlussfolgerung

Die Anwendung von CPM im Rahmen eines komplexen Behandlungsprogramms in einer Anschlussheilbehandlung nach Implantation von Schulterendoprothesen kann zu einer Verbesserung in der Beweglichkeit und zu einer Reduktion der Schmerzmitteleinnahme beitragen. Es zeigten sich Hinweise auf einen positiven Effekt, insbesondere während der frühen Phase der Rehabilitation. Aufgrund des Studiendesigns können die Ergebnisse zwar keinen Kausalitätsnachweis erbringen, aber vor allem hypothesengenerierend betrachtet werden. Ob durch den Einsatz von CPM Vor- oder Nachteile gegenüber einer Behandlung mit physiotherapeutischer Mobilisation oder eines rein aktiven Übungsprogramms bestehen, sollte daher in gezielten prospektiven Untersuchungen erhoben werden. Inwieweit hier auch wirtschaftliche Vorteile zu beachten sind, sollte ebenfalls in derartigen Untersuchungen geprüft werden.

## Data Availability

Die Daten, die die Ergebnisse dieser Studie stützen, sind aus Gründen der Sensibilität nicht öffentlich zugänglich und können auf begründete Anfrage beim korrespondierenden Autor angefordert werden.

## Abkürzungen

AHB: Anschlussheilbehandlung
CPM: Continuous Passive Motion
NSAR: Nichtsteroidales Antirheumatikum
NRS: Nummerische Rating Skala
